# Engineering of Live Chimeric Vaccines against Human Metapneumovirus

**DOI:** 10.3390/pathogens9020135

**Published:** 2020-02-19

**Authors:** Daniela Ogonczyk Makowska, Marie-Ève Hamelin, Guy Boivin

**Affiliations:** Centre de Recherche en Infectiologie of the Centre Hospitalier Universitaire de Québec and Université Laval, Québec, QC G1V 4G2, Canada; daniela.ogonczyk-makowska@crchudequebec.ulaval.ca (D.O.M.); marie-eve.hamelin@crchudequebec.ulaval.ca (M.-È.H.)

**Keywords:** human metapneumovirus, respiratory syncytial virus, chimeric vaccines, recombinant vaccines, viral vectors, bivalent vaccines

## Abstract

Human metapneumovirus (HMPV) is an important human pathogen that, along with respiratory syncytial virus (RSV), is a major cause of respiratory tract infections in young infants. Development of an effective vaccine against Pneumoviruses has proven to be particularly difficult; despite over 50 years of research in this field, no vaccine against HMPV or RSV is currently available. Recombinant chimeric viruses expressing antigens of other viruses can be generated by reverse genetics and used for simultaneous immunization against more than one pathogen. This approach can result in the development of promising vaccine candidates against HMPV, and several studies have indeed validated viral vectors expressing HMPV antigens. In this review, we summarize current efforts in generating recombinant chimeric vaccines against HMPV, and we discuss their potential optimization based on the correspondence with RSV studies.

## 1. Introduction

Acute respiratory tract infections (RTIs) are the 5^th^ leading global cause of mortality among all age groups and the 3^rd^ leading mortality cause in children younger than 5 years old [1]. An important number of RTIs is caused by viruses belonging to the Pneumoviridae family, namely, respiratory syncytial virus (RSV) and human metapneumovirus (HMPV) that are responsible for approximately 31% and 5.5% of RTIs in children, respectively [2]. 

HMPV is a single-stranded, negative-sense RNA virus belonging to the family of Pneumoviridae, genus Metapneumovirus. The genus has two species: human and avian metapneumovirus (AMPV). HMPVs are divided into two major genetic subtypes: A and B that are further divided into sublineages A1, A2a, A2b, B1, and B2 [3]. Lineages A and B share 80% and 90% of their nucleotide and amino acid sequence identity, correspondingly [4]. The major antigen of HMPV, the F protein, is highly conserved and a high level of cross-neutralization and cross-protection is observed between the two lineages [5,6]. The clinical signs of HMPV disease do not vary significantly between the different genetic lineages of HMPV [7]. AMPV, previously known as turkey rhinotracheitis virus, is an important poultry pathogen, divided into subgroups A, B, C, and D [8,9,10]. The subgroup C (AMPV_C) shares 80% of the amino acid identity of N, P, M, F, M2-1, and M2-2 proteins with HMPV [11], making it the closest related virus to HMPV. 

HMPV is a relatively newly-discovered pathogen, described for the first time by the researchers from the Netherlands in 2001 [11]. Its late identification can be explained by the similarity of symptoms caused by HMPV and RSV, as well as the difficulty to observe HMPV growth in vitro. Both viruses cause pneumonia and bronchiolitis, with variable severity of illness according to age [12]. Although HMPV disease is generally less severe compared to RSV, the incidence of infections caused by HMPV is similar to that of influenza and parainfluenza, and it causes a significant number of hospitalizations every year [13]. Infections with either virus are associated with the development of asthma and its exacerbations, yet the causal relationship has not been established [2,14,15]. Although both pneumoviruses can infect any age group, they cause the greatest disease severity in young infants. HMPV infects mainly children between 6 and 12 months of age, whereas RSV infects infants earlier, within the first 2–3 months after the birth [2,16,17,18,19]. Virtually every child is infected by RSV by the age of 2 [20] and by HMPV by the age of 5 [21], and the incidence of reinfections with both viruses increases significantly after the age of 50 causing a lot of RTIs in elderly [19,22]. Another population at risk of HMPV infections is comprised of immunosuppressed patients [23]. 

### 1.1. Need for a Vaccine

Despite the high frequency of pneumoviral infections and over 50 years of research in this field, no licensed vaccine against HMPV or RSV is currently available. Among the numerous vaccine candidates against RSV that have been developed, only a few have advanced to clinical trials and most of them failed as a result of insufficient immunogenicity or underattenuation [24]. The only anti-HMPV vaccine advanced to clinical trials until now—a live-attenuated recombinant rHMPV-P_A_ virus, in which HMPV-P protein was exchanged for its counterpart from AMPV_C—proved to be insufficiently immunogenic and overattenuated in seronegative children, thus leaving no advanced candidates for an anti-HMPV vaccine [25]. 

This lack of effective vaccine candidates against HMPV can be explained by the recent discovery of the virus, but also by the lack of a successful vaccine against closely related RSV that could serve as a base for vaccine design. One of the reasons for the slow progress in this field is the clinical failure of formalin-inactivated RSV-vaccine (FI-RSV) that occurred in the 1960s. The administration of FI-RSV led to the development of an exaggerated immune response to wild type (wt) RSV infection with enhanced pulmonary disease (EPD) and two vaccinated children died [26]. EPD has been further documented in various animal models, indicating that it is not only a human phenomenon [27,28,29,30]. A similar effect was observed for a formalin or heat-inactivated HMPV vaccine, which induced the symptoms of EPD in rodents [31,32]. Another reason for slow vaccine development is the transient immunity provided by natural infection with RSV and HMPV. As documented in RSV-seropositive adults, levels of RSV-neutralizing antibodies correlate with the resistance to subsequent RSV infection, but the protection they confer is incomplete and short-lasting, which results in frequent reinfections [20,33]. The immune response to primary HMPV infection was described to be weak and aberrant in BALB/c mice, with excessive Th2-cytokines production at later stages of infection, which correlated with airways hyperresponsiveness and the development of asthma [34]. Pneumoviruses have also developed many mechanisms to evade immune responses. The RSV nonstructural proteins NS1 and NS2 suppress the IFN-induced antiviral response in infected cells and removal of these proteins resulted in the induction of high levels of IFN alpha and beta in vitro [35,36,37]. HMPV shares with RSV the same ability to decrease IFN response, despite the lack of NS1 and NS2 proteins. It has been documented that HMPV-G protein has the ability to inhibit the IFN type 1 response in vitro and in vivo [38,39] and that its SH and M2-2 proteins can also modulate the host’s immune responses [40,41,42]. Both HMPV and RSV can persist in the lungs of an infected animal host, despite the presence of neutralizing antibodies [34,43]. Such persistence has been documented by RT-PCR detection of viral RNA in the guinea pig and mouse models for RSV [44,45,46] and in mice for HMPV [43]. Another hurdle to effective immunization is the presence of maternal antibodies in the bloodstream of vaccinated infants that can impair the immunogenic effect of the vaccine [47], yet the role of maternal antibodies in RSV infection is not fully understood [48]. 

### 1.2. Vaccine Development 

The main objectives in pneumovirus vaccine development are to avoid EPD and obtain sufficient immunogenicity without causing disease. Viral protein antigens can be used as subunit vaccines to induce an appropriate immune response. These proteins can be delivered as nanoparticles, virus-like particles (VLPs), or they can be coupled with adjuvants [49]. Another interesting strategy is to immunize with viral nucleic acid coding for viral antigens, as it has been documented for RSV and HMPV vaccine candidates [50,51,52]. An mRNA-based vaccine coding for the F proteins of HMPV and human parainfluenza type 3 virus (PIV3) has been recently advanced to phase 1 clinical trial [53]. 

Among the three surface proteins of HMPV (F, G, and SH), the F protein constitutes the major HMPV antigen [6,54,55]. Although immunogenic, its G protein does not induce a potent protective immune response [54,56] and is not indispensable for viral replication in vivo [57]. HMPV-SH protein was shown not to confer any significant protection [54], the same being observed for RSV [58]. The F protein is also the major antigen of RSV, more immunogenic than the G protein [59], although the latter is also able to induce a protective immune response [60,61] and has been frequently tested as a subunit vaccine [62,63,64]. RSV-F protein stabilized in its pre-fusion form by introducing disulfide bond (DS) and cavity-filling (Cav1) mutations (DS-Cav1) is currently being tested as a subunit vaccine in phase I clinical trials (ClinicalTrials.gov Identifier: NCT03049488). The pre-fusion RSV-F was shown to be more immunogenic than its postfusion conformation, as a result of an exposition of a unique antigenic site Ø that is recognized by very potent RSV-neutralizing antibodies [65,66,67]. Trials with the HMPV-F subunit vaccine showed that the vaccine was immunogenic, but not protective in a rodent model [68]. To increase its immunogenic potential, HMPV-F protein has been stabilized in its pre-fusion state by analogous strategies as for RSV-F, but its immunogenicity was not enhanced [69,70]. This difference between pre-fusion RSV-F and HMPV-F can be explained by additional glycosylation at the apex of the pre-fusion form of HMPV-F. HMPV-F and RSV-F share some antigenic sites, and antibodies able to cross-neutralize the two pneumoviruses were identified [71,72,73,74,75]. Grafting of a major protective epitope from RSV-F into HMPV-F protein resulted in the elaboration of a chimeric protein carrying epitopes of both pneumoviruses, an interesting candidate for vaccine design [76,77].

Several promising nanoparticle vaccines against RSV have been elaborated [78,79], among which is ResVax, a nanoparticle RSV-F-based vaccine developed by Novavax [80]. ResVax has been recently tested in phase 3 clinical trials in maternal immunization model of lower RTIs prevention in infants (NCT02624947), where it did not meet its clinical endpoint [81]. VLPs are structures composed of the proteins of viral capsid; they do not contain the viral genome and are replication-incompetent. Coexpression of HMPV-F, -G, and -M proteins leads to the formation of VLPs in vitro [82], yet the expression of only F and M proteins has been shown sufficient for VLPs assembly [83]. Several potential VLPs vaccines against HMPV [82,83,84,85], and many more against RSV [78,86,87,88,89,90,91,92,93,94,95,96,97,98,99,100,101,102,103,104,105], were developed, but none of them has been advanced to clinical trials. A promising vaccine candidate—a VLP composed of pre-fusion, postfusion RSV-F, or both together, assembled with HMPV-M protein—conferred full protection against RSV infection in immunized mice [96]. 

On the other hand, immunization with an attenuated replication-competent virus is a very promising approach. In this case, it is possible to obtain a vaccine that presents all viral epitopes and is able to induce both humoral and cellular immune responses [106]. The main disadvantage of live attenuated vaccines is the risk of reversion of the attenuated profile, restoration of infectivity and subsequent development of the disease [107]. Live attenuated viruses can be divided into two groups: non-recombinant and recombinant or chimeric viruses. Non-recombinant viruses are rendered less infectious due to genetic modifications that appear naturally during serial passages in vitro in conditions of environmental stress. Several attenuated strains of RSV and HMPV have been obtained as a result of serial cold passages or chemical mutagenesis, which confer a temperature-sensitive (*ts*) phenotype to a virus [108,109,110,111,112]. Apart from the mentioned selection strategy, recombinant attenuated viruses can be generated by reverse genetics, a technique that makes it possible to generate functional particles of a modified virus based on its genetic material [113,114]. This approach allows not only to introduce attenuating mutations directly into the viral genome [57,115], but also to express exogenous antigens in the backbone of the virus, leading to the development of polyvalent, chimeric vaccines. The insertion of a foreign gene can have a potentially attenuating effect on its own, for example, by rearranging the order of genes in terms of 3’-5’ expression gradient, as it is observed in paramyxoviruses. Gene order can also be changed in a more invasive way by moving virulence genes from the high-expression position to the low-expression locus. Alternatively, the replication efficiency can be decreased by deletions or silencing of nonessential viral proteins that play an accessory function in the life cycle of the virus. It is also possible to swap some of the genes between strains of different host preferences, thus introducing new host range restrictions. HMPV virus with the P gene exchanged for its AMPV_C counterpart (rHMPV-P_A_) was more attenuated than wt HMPV, and yet protective against HMPV infection in African green monkeys (AGMs) [116]. In this review, we will focus on and discuss in detail the development of live recombinant vaccines against HMPV.

### 1.3. Target Populations 

The ultimate goal of HMPV vaccination is the prevention of lower RTIs in populations at risk. Similarly to RSV, the target populations for HMPV vaccination are young children, elderly people, and pregnant women [117]. Early vaccination of infants and young children can potentially prevent HMPV-infections and the transmission of the virus. Replication-competent vaccines, namely, live-attenuated HMPV or recombinant viruses are a good choice for this population. Another vaccine strategy, prime-boost regimen, consists of vaccinating with gene-based/live vaccine first and then with a protein/particle-based vaccine [118]. Immunization of pregnant women can not only provide a passive antibody transfer to their children but also prevent a mother-to-child transmission of HMPV. Live vaccines are considered being too risky to be used in this population; therefore, subunit vaccines or VLPs with standard adjuvants should be considered. The effect of vaccination of adult populations with live vaccines can be hampered by the presence of anti-HMPV antibodies in the bloodstream or in the respiratory tract (RT). For the vaccination of elderly people, who had experienced multiple HMPV infections, subunit or VLPs with a potent adjuvant are recommended [118]. 

## 2. Vector-Based Chimeric Vaccines

One of the strategies aimed to circumvent the problem of incomplete immunity provided by natural HMPV infection is to express its protective antigens in the backbone of a more immunogenic virus. The increase in immunogenicity can also be provided by improved antigen expression by the vector, as demonstrated for a recombinant bovine/human PIV3 (rB/HPIV3) expressing either RSV-F or G proteins [60] and for human parainfluenza type 1 virus (HPIV1) expressing HMPV antigens [54]. HMPV is difficult to grow in cell culture; vectoring its antigens with the backbone of a better-replicating virus can facilitate vaccine development and manufacturing. For instance, rB/HPIV3 expressing RSV-F reaches 10–100-fold higher viral titers in vivo compared to wt RSV [60] and replicates more efficiently in the RT of AGMs than wt RSV or HMPV [119,120]. Expression of a foreign antigen in the backbone of another virus can also mitigate the problem of pre-existing immunity that often diminishes the effect of the vaccine. Using vectors of another host range can facilitate the immunization of seropositive individuals with no risk of causing disease, as it has been described for Newcastle Disease Virus (NDV) expressing RSV-F. The majority of live vaccines against RSV and HMPV up to date have been attenuated by a few codon changes in their genome. Therefore, using more stably attenuated backbone can render the attenuation more reliable, and decrease the risk of the reversion of an attenuated phenotype. The insertion of an additional gene can itself influence the virus’ replicative capacity, thus limiting its ability to spread and cause the disease, but also to create the risk of overattenuation. This subtle balance between attenuation and immunogenicity remains the major challenge of vaccine development. 

In general, vector-based chimeric vaccines retain the advantages of live attenuated vaccines and they can be readily generated by reverse genetics. Most of the backbones used so far for vectoring pneumoviral antigens belong to the Paramyxoviridae family. These viruses are closely related, both phylogenetically and structurally, to pneumoviruses. This close relationship increases the chance of an efficient expression and integration of a pneumoviral protein in the background of a paramyxoviral vector. Among paramyxoviruses, the most widely used vaccine backbones are bovine parainfluenza virus type 3 (BPIV3) and its recombinant derivative rB/HPIV3, HPIV1, parainfluenza virus type 5 (PIV5), Newcastle Disease Virus (NDV), and Sendai virus (SeV). The reasons explaining this frequent use of paramyxoviruses as vaccine backbones are numerous: First, their genomes are well-characterized and complete genomic sequences of all known members of this family are easily accessible [121]. Second, their genomes are simple and organized in a modular way. Tandem alignment of the genes, of which the most are transcribed as separate mRNA products, facilitates genetic manipulations [122]. Third, most paramyxoviruses are able to replicate efficiently in cell lines certified for vaccine manufacture, such as Vero cells, which can facilitate vaccine development [122]. Fourth, as they replicate entirely in the cytoplasm and their replication cycle does not involve the integration into the host cell genome, their use is potentially safe [121]. Also, recombination events between mononegaviruses have not been observed in nature and they remain extremely rare in vitro, even in optimized conditions [123,124], indicating that there is a low probability of gene exchange between engineered vaccine viruses and the pathogens present in the environment. Fifth, the small probability of recombination also contributes to the stability of a genetic insert, providing a stable foreign gene expression system. Paramyxoviruses are able to stably express several exogenes simultaneously and the level of expression can be manipulated by changing the position of gene insertion [125]. Sixth, most paramyxoviruses infect their host via their RT, representing an easy and safe route of administration of the vaccine as well as for induction of both local and systemic immune responses. Last, but not least, there are many animal paramyxoviruses that are naturally attenuated in humans due to a host range restriction. Mutations that render these viruses harmless to people have been identified and characterized, thus making attenuation of different paramyxoviruses possible [122]. 

## 3. Recombinant Virus Engineering

### 3.1. Reverse Genetics

**Figure 1:**
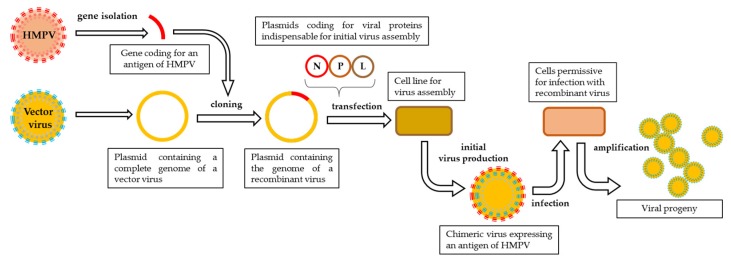
Schematic representation of reverse genetics pipeline. The exogene of interest is cloned into a plasmid containing a complete genome of a vector virus and then transfected, along with satellite plasmids coding for viral proteins indispensable to initiate viral assembly, into a cell line designed for initial virus production. The first progeny of the recombinant virus is harvested and propagated on a permissive cell line.

Negative strand viruses can be readily recovered from cell cultures by means of reverse genetics [126]. This technique makes it possible to engineer a fully functional virus starting from its genetic sequence. The genetic material in the form of cDNA can be easily modified according to the vaccine design. The technique is based on transfecting permissive cells with a plasmid coding for the viral genome and satellite plasmids coding for all the proteins necessary for the formation of a ribonucleoprotein complex (RNP) that initiates the transcription of viral genes (Figure 1). The proteins indispensable to form the RNP in paramyxoviruses and pneumoviruses are N, P, and L proteins [127,128,129], yet the addition of M2-1 protein facilitates the recovery HMPV from cDNA [130]. The elaboration of a polyvalent vaccine can be accomplished by either cloning additional genes into the plasmid coding for viral genome or replacing protective antigens of a vector with the ones of another pathogen.

### 3.2. Genomic Organization of Paramyxoviridae and Pneumoviridae

**Figure 2:**
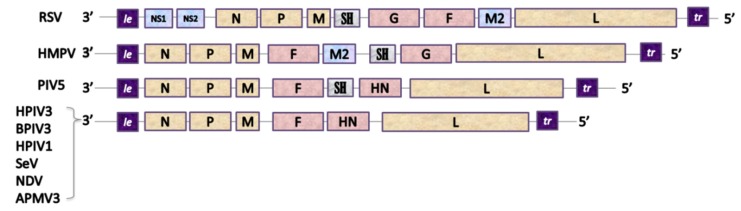
Genetic organization of Pneumoviruses and some of Paramyxoviruses used as viral vectors. *Pneumoviridae*: RSV: Respiratory Syncytial Virus (*Orthopneumovirus*), HMPV: Human Metapneumovirus (*Metapneumovirus*). *Paramyxoviridae*: PIV5: Parainfluenza type 5 virus (*Rubulavirus*), HPIV3: Human Parainfluenza type 3 virus, BPIV3: Bovine Parainfluenza type 3 virus, HPIV1: Human Parainfluenza type 1 virus and SeV: Sendai Virus (*Respirovirus*), NDV: Newcastle Disease Virus and APMV3: Avian Paramyxovirus type 3 (*Avulavirus).* Genes: le: leader, NS1 and NS2: accessory proteins of RSV, N: nucleoprotein, P: phosphoprotein, M: matrix protein, F: fusion protein, SH: small hydrophobic protein, G: attachment glycoprotein, HN: Haemagglutinin-Neuraminidase protein, M2: gene coding for M2-1 and M2-2 proteins, L: large polymerase subunit.

The genomes of Paramyxoviridae and Pneumoviridae consist of a simple, nonsegmented, linear, single-stranded, 15,000–19,000-nucleotide-long negative RNA that contains 6–10 genes [121]. The most 3’-proximal region consists of a *leader* (le)—a 50-nucleotide-long promoter—and each gene is preceded and followed by a short (10–13 nucleotides) conserved sequence named *gene start* (GS) and *gene end* (GE). These sequences act like transcription control signals for viral RNA-dependent RNA-polymerase (vRNAP) and they guide the enzyme along the genome [131]. Single genes are separated from each other by short, non-coding intergenic regions and the order of the genes is usually conserved as 3’-Nucleoprotein (N), Phosphoprotein (P), Matrix Protein (M), Glycoprotein (G), Hemagglutinin-Neuraminidase (HN), or Large Polymerase subunit (L)–5’ with the presence and location of additional genes depending on the virus [132] (Figure 2). 

The 5’-end of a genome contains a trailer sequence (*tr*) of a variable length, ranging from 50 up to 707 nucleotides [121,133]. The genomic RNA of paramyxoviruses and pneumoviruses does not exist as an unbound RNA-particle: it is always assembled with numerous copies of N protein and forms a helicoidal nucleocapsid. In paramyxoviruses, each N protein molecule is associated with precisely six nucleotides, a feature that is believed to underlay the ‘’rule of six’’, i.e., the length of the genome of paramyxoviruses has to be a multiple of six for an effective viral replication [134,135]. The stringent adhesion to this rule is observed for SeV [136]; for other viruses, like PIV5, NDV, and HPIV3, it strongly increases the efficacy of replication [137,138,139]. The fact that it does not give any replicative advantage to RSV [140] might be explained by distinct differences in nucleocapsid structure of the two families [141]. Transcription of the viral genome is initiated at the 3’-end and a 3’-to 5’ expression gradient is observed. The complex of vRNAP sporadically fails to resume the synthesis of another distinct mRNA at each gene junction, which results in a gradual loss of transcription-efficacy along the genome [121].

### 3.3. Principles of Exogene Insertions

**Figure 3:**
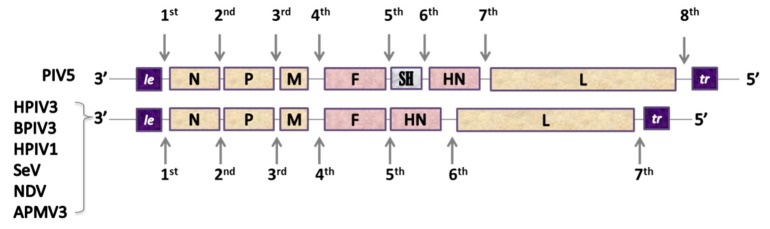
Insert-positions in the paramyxovirus genomes.

**Figure 4:**
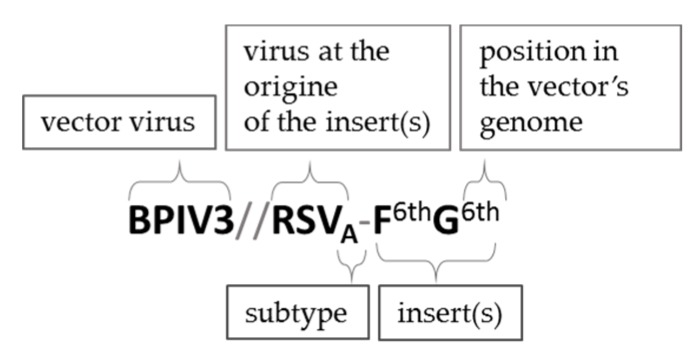
The nomenclature used in this review to describe chimeric viruses expressing additional antigens. The inserts are marked according to their 3’-5’ order of the vector’s genome. The subtype of the virus at the origin of the insert is marked if it is specified in the source. Although discussing different chimeric viruses based on the same vector that are mentioned in the same study, recombinant viruses can be also referred to as F^1st^, G^2nd^, etc. constructs. The nomenclature of vector backbones modified by additional mutations, protein swapping, etc. (i.e., rHPIV1-C^Δ170^, rHMPV-P_A_) was unchanged in relation to the source publication.

The main principles to be taken into consideration while engineering the genome of recombinant paramyxoviruses are the preferential adherence to the rule of six, a coherence of transcription control signals (GS/GE) used to drive the expression of a foreign antigen and exogene positioning in the transcription gradient. As it has been documented in numerous studies, the GS and GE signals of the vector virus can efficiently direct the expression of an exogenous protein [60]. Although GS and GE signals are often highly conserved along the viral genome, there might be variations in their sequences and transcription efficacy [142]. Flanking the sequence of GFP inserted into the 6^th^ genome position of PIV5 with GS/GE specific for either 2^nd^ or 7^th^ gene junctions resulted in large differences in GFP expression levels (Figure 3) [143]. GS/GE characteristics for the 1^st^ junction provided better expression levels than the ones originating from the 7^th^. It is therefore important to flank the exogene with potent transcription regulators. In accordance with the 3’-5’ transcription gradient, exogenes placed in more 3’-proximal position should be expressed better than 5’-proximal inserts, yet the tendency cannot be described as linear and some deviations are observed [144]. The adherence to this gradient might also be influenced by the type of attenuation of vector virus, as it has been demonstrated for HPIV1 bearing RSV-F protein [145]. The positions of an exogene in the viral genome are described in Figure 3 and the nomenclature used to label chimeric viruses in this review is explained in Figure 4. 

On one hand, a 3’-proximal insertion should provide the best level of expression; on the other hand, it can influence the level of transcription of all downstream genes, leading to a decrease in viral replication. RSV-F insertion into 1^st^ position of rB/HPIV3 genome reduced the expression of downstream genes by 20–45% [146]; 2^nd^ genomic position can provide a good expression, but it can also influence the N:P protein ratio, which plays a decisive role in the replicative capacity of paramyxoviruses, causing an additional attenuating effect [146]. Insertions in either 1^st^ or 2^nd^ genomic positions are the most privileged for PIV3-based vectors, with the 2^nd^ position usually providing better virus recovery, higher viral replication, and better exogene expression [147]. For some vectors, namely, NDV and Avian paramyxovirus serotype 3 (APMV3), the 3^rd^ position is the most advantageous, whereas the insertion at the 2^nd^ one results in delayed viral replication and the largest reduction in virus recovery [148,149,150]. The 4^th^ and the 5^th^ positions are not frequently used, so as to not influence the expression of vector’s surface proteins (F and HN) [146], with the exception of studies on SeV bearing either RSV-F or HMPV-F at the 5^th^ position [151,152,153]. 

The nature of the insert itself can also influence the vector’s biology. The rB/HPIV3//RSV-F^1st^ virus showed an 8-fold reduced replication in vitro compared to rB/HPIV3, whereas the RSV-G^1st^ insert did not influence viral replication [60]. This decrease in viral growth might have been due to excessive syncytia formation and increased cytopathology resulting from the expression of a second fusion protein. Another reason might have been the size of the insert–a bigger F protein might have influenced the replication more significantly than a smaller G protein. The size of an exogene can significantly change the replicative capacity of the virus, as it has been shown for HPIV3 vector bearing inserts of different sizes [154]. The level of integration of a foreign protein into a vector’s particle can also be a price to pay in the exchange for efficient expression of the exogene, as described in Chapter 5 of this review. Improved exogene integration into the vector’s backbone can be obtained by the substitution of the transmembrane domain (TM) and cytoplasmic tail (CT) of the inserts with their equivalents from the vector virus. As demonstrated for HPIV1 vector bearing RSV-F protein either in its wt or chimeric TMCT form, packaging of RSV-F^(TMCT)^ was strongly improved, but the chimeric virus was overattenuated and not protective in hamsters [155]. 

## 4. Examples of Chimeric Anti-HMPV Vaccines

### 4.1. Recombinant Bovine/Human Parainfluenza Type 3

Replacing the F and HN genes of BPIV3 by their HPIV3 counterparts resulted in the elaboration of rB/HPIV3, a recombinant virus broadly used for expressing foreign antigens. The virus is more immunogenic in humans than BPIV3 while retaining its attenuation profile [156]. Its safety and immunogenicity have been documented in adults, HPIV3-seropositive, and HPIV3-seronegative children [157]. rB/HPIV3 reaches 10–100-fold higher titers in vivo than RSV, which makes it a good platform for the studies on anti-pneumoviral vaccines [60]. PIV3 genome is approximately 15 kb in length and includes six structural genes [121]. Both HPIV3 and BPIV3 adhere to the “rule of six”; several viruses that were recovered from genome cDNA whose length did not fulfill this condition were found to have accommodated nucleotide insertions correcting their genome length [125]. 

rB/HPIV3 was broadly used to express RSV or HMPV antigens and the examples of rB/HPIV3-based vaccines that were tested in preclinical trials are shown in Table 1. Many different positions were tested for RSV-F insertion and a 30–69-fold gradual decrease in exogene expression was observed between the 1^st^ and the 6^th^ one [146]. Although 2–3 times more attenuating than the 3^rd^ or 6^th^ position, the 2^nd^ one provides a very attractive attenuation/exogene expression ratio. rB/HPIV3 virus bearing RSV-F in the 2^nd^ position (MEDI-534) developed by MedImmune was tested in phase 1/2 clinical trials (NCT00686075), but it was proven insufficiently immunogenic [158,159,160]. rB/HPIV3 can accommodate an insert of both RSV-F and G proteins in the 1^st^ position with little decrease in replication in vitro [61]. The same double insert placed in the 5^th^ position resulted in a *ts* phenotype and restricted replication in vivo, yet the virus was immunogenic and protective in hamsters [161]. 

The insertion of HMPV-F gene into either 1^st^ and 2^nd^ positions of rB/HPIV3 genome resulted in efficient exogene expression as integral membrane proteins [147]. HMPV-F^1st^ chimeras were more difficult to recover and replicated less efficiently, and the same was observed for RSV-F inserts. The viruses were immunogenic and protective in hamsters and the HMPV-F^2nd^ virus was subsequently evaluated as a vaccine in African green monkeys (AGMs) [120]. The virus replicated better than wt HMPV and it was protective against wt HMPV challenge. rB/HPIV3//HMPV-F^2nd^ induced effective anti-HMPV-specific T-cell responses, that were slightly lower compared to wt HMPV. The levels of antibodies induced by the chimeric virus correlated with those that provided protection against lower respiratory tract (LRT) infections in primates [162] and infants [163]. 

### 4.2. Human Parainfluenza Type 1

Human parainfluenza type 1 (HPIV1) is the major cause of croup, and it is responsible for approximately 38% of HPIV infections in children before the age of 5 [164]. As it is an important pediatric pathogen with no protective vaccine available, the use of this virus as a backbone for a bivalent vaccine is highly warranted. HPIV1 shares the same genomic organization with other HPIV viruses. Recombinant HPIV1 tested as a vaccine in preclinical studies in AGMs (rHPIV-C^R84G/Δ170^HN^553A^L^Y942A^) has been attenuated by mutations in its P/C, HN, and L genes [165]. Tested as a vaccine in HPIV1-seropositive and HPIV1-seronegative children, it was shown to be well-tolerated but overattenuated [166]. Considering the additional attenuating effect of exogene insertion, subsequent studies concentrated on HPIV1 bearing just a single attenuating mutation—either C^Δ170^ or L^Y942A^. The examples of HPIV1- based vaccines are shown in Table 2. 

Mackow et al. tested rHPIV1-C^Δ170^ and rHPIV1-L^Y942A^ vectors for expression of RSV-F at the 1^st^, 2^nd^, or 3^rd^ genomic positions [145]. They showed that rHPIV1-L^Y942A^ backbone is overattenuated upon RSV-F insertions and that the rHPIV1-C^Δ170^ backbone is a more suitable vector. rHPIV1-C^Δ170^ has been subsequently tested to express either native RSV-F or chimeric RSV-F^(TMCT)^ protein from the 1^st^ and the 2^nd^ positions [155]. Wild type HPIV1 has been tested as a vector for expression of the F, G, and SH surface proteins of HMPV to examine their relative contribution to inducing HMPV-specific antibodies [54]. Contrary to G^3rd^ and SH^3rd^ insertions, F^1st^ insertion impaired virus growth and the expression of the vector’s HN gene. Vaccination with HPIV1//HMPV-F^1st^ provided significant protection against wt HMPV challenge, but even two doses were less protective than a single immunization with wt HMPV. This study showed that the HMPV-F is the major viral antigen, being more immunogenic than HMPV-G or SH [54]. The HPIV1 backbone was also used to express the F protein of HMPV_A and evaluate its ability to induce cross-protection against HMPV_B [6]. This study showed that the F protein of HMPV_A can induce cross-protection against a heterologous HMPV strain and that the HPIV1 backbone is suitable for vectoring HMPV antigens. 

### 4.3. Sendai Virus

Sendai virus (SeV) is a murine parainfluenza type 1 virus. Its natural host range restriction provides safety for humans, with no risk of reversion of the attenuated phenotype and it is capable of inducing durable and strong immune responses [167]. SeV is closely related to HPIV1 and has been developed as a heterologous vaccine that increases protection from HPIV1 infections in animal models and in humans [168,169,170]. The length of the SeV genome is of approximately 15.3 kb and the virus strictly adheres to the rule of six for efficient replication [171]. 

SeV has been shown to accommodate and stably express inserts up to 3.2 kb in size with the replication rate inversely correlated with the total genome length [172]. It has been broadly used as a backbone for expressing RSV-F or G proteins from its 5^th^ genomic positions and tested as a vaccine alone [151,152,173,174,175] or in formulations of multiple recombinant SeV expressing the antigens of different HPIV types [176,177]. The 5^th^ genomic position was also used for the expression of HMPV-F [153]. The virus was recovered by reverse genetics, although there was a deletion in the exogene’s sequence that resulted in expression of a truncated HMPV-F protein. It was 303 amino acids in length, retaining the F2 part (comprising of a signal peptide, fusion peptide and heptad repeat A) and a fragment of the F1 part, lacking Heptad Repeat B and both TM and CT domains. Although truncated, the protein was immunogenic and conferred protection against wt HMPV_A1 and A2 in immunized hamsters. This study showed that vectoring HMPV antigens with SeV is a promising strategy and that a soluble form of the HMPV-F protein remains immunogenic [153]. The examples of these chimeric SeV are shown in Table 3.

### 4.4. Newcastle Disease Virus

Newcastle disease virus, an avian paramyxovirus serotype 1 (APMV1) virus, is an important poultry pathogen that recently drew a lot of attention for its potential use both as an oncolytic agent and as a vaccine vector. Although it is recognized as an avian pathogen, it has been experimentally proven to infect several mammal species including mice, hamsters, guinea pigs, rabbits, ferrets, calves, pigs, and non-human primates. Natural NDV infections are very rare in humans; the cases of infection have been documented in bird handlers exposed to the virus [178,179] and no human-to-human transmission has ever been observed [180]. Various NDV strains are classified as low virulent (lentogenic), mildly virulent (mesogenic), or highly virulent (velogenic). Concerning the high contagiousness of NDV and its potentially detrimental impact on the poultry industry, lentogenic strains of NDV were classified as biosafety level 2 (BSL-2) pathogens, with mesogenic and velogenic strains being BSL-3 pathogens [181]. Some lentogenic strains, namely, LaSota and B1, are used as naturally-attenuated vaccines to protect the poultry. Although mesogenic NDV strains are not frequently used as vaccine vectors, a mesogenic strain Beaudette C (BC) tested as a vaccine vector for the HPIV3-HN protein in non-human primates was well-tolerated and more immunogenic compared to the LaSota strain [182]. 

Similarly to SeV, NDV is safe in humans due to a natural host range restriction with no presence of pre-existing immunity. It is highly immunogenic, inducing a potent IFN type 1 response [183]. Its genome is approximately 15.2 kb in length and the virus adheres to the rule of six for efficient replication [180]. The virus can accommodate exogenes up to 4.5 kb in length and it can effectively express three additional proteins [184]. An exogene can be placed at the gene junction between any two genes of the virus, yet the 3^rd^ position has been found to be the most optimal for NDV vector [149,182,185,186] with the 1^st^ and the 2^nd^ ones being the least optimal [148,149]. NDV has been used for vectoring the antigens of RSV [185], AMPV [187,188,189], HIV [149,190,191], HPIV3 [182], influenza A virus [192], SARS-coronavirus [186], Nipah virus [193], and Ebola virus [194]. Examples of NDV vectoring pneumoviral antigens are reported in Table 4.

Although NDV has not yet been used as a vector for HMPV antigens, it was found to be an efficient backbone for surface proteins of AMPV_C. LaSota NDV strain has been used for vectoring the G protein of AMPV_C and tested as a bivalent vaccine against NDV and AMPV_C in turkeys [187]. The NDV//AMPV_C_-G^5th^ virus conferred partial protection in 50% of vaccinated birds, indicating that AMPV_C-G protein alone is not sufficient to induce protective immunity. The immunogenicity of this construct was strongly improved when AMPV_C-F and G proteins were expressed together from the 5^th^ genomic position of NDV genome [189]. Similarly to HMPV, the F protein of AMPV_C is indispensable to induce a potent immune response in a vaccinated host. 

### 4.5. Vesicular Stomatitis Virus

**Figure 5:**
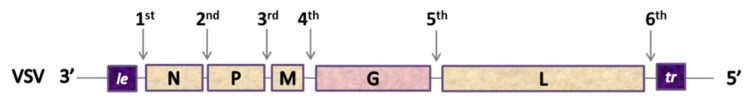
The organization of the genome of VSV with the indication of insert positions.

Vesicular stomatitis virus is a pathogen of horses and livestock, belonging to the Rhabdoviridae family. Rhabdoviridae and Paramyxoviridae both belong to the same order of negative-strand viruses, Mononegavirales. The genome of VSV is a single-stranded, nonsegmented RNA of negative polarity of approximately 11.1 kb in length and, due to the simplicity of its genome, it was used as a model to study the transcription and replication of nonsegmented negative-stranded viruses (NNSV) (Figure 5) [121].

VSV shares the same advantages as a viral vector with paramyxoviruses, although with superior growth in vitro [195]. It provides a good level of protein expression: Chloramphenicol acetyltransferase protein (CAT) expressed from the 5^th^ position of VSV genome constituted 1.7% of all the proteins produced by rVSV-infected cells [195]. The expression of VSV genes follows the polar gradient observed for paramyxoviruses, and 3’-proximal insert positions influencing N:P proteins ratio, can be detrimental for virus replication. An N:P ratio between 1:1 and 2:1 has been shown optimal for viral replication, whereas a ratio below or above this value was associated with decreased replicative capacity of the virus [196]. 

VSV vector has two potential drawbacks, namely, neurotoxicity mediated by its G protein [197,198] and induction of a potent VSV-neutralizing antibody response specific to its G protein even upon a single immunization, which makes the boost with a heterologous vaccine impossible and limits the future use of VSV-based vaccines for the vaccinated patient [199]. This neurotropism results in significant viral replication in brain and mortality in mice infected by VSV [200], raising some serious concerns about using this virus as a vaccine vector. Although VSV is not a human pathogen, some human infection can occur as a result of contact with infected animals [201,202,203]. One case of encephalitis caused by VSV infection was reported in a 3-year-old boy from Panama [204]. Because of significant morbidity in VSV-infected cattle, VSV has been included in the list of potentially harmful agents by the United States Department of Agriculture [205]. 

To circumvent the problem of G-mediated VSV neurotoxicity, an attenuated VSV in which the G protein had been exchanged for the GP protein of lymphocytic choriomeningitis virus (LCMV), called rVSV-GP, has been designed [206]. This chimeric virus was used to express the RSV-F protein at the 5^th^ genomic position in three variants: native, codon-optimized, and codon-optimized with TMCT domain exchange [207]. Efficient expression of native RSV-F and RSV-G proteins from the 5^th^ and the 4^th^ genomic positions, respectively, has been also documented for rVSV, but the viruses were not tested in vivo [208]. RSV-F^5th^ constructs were also tested in the context of a VSV backbone attenuated as a result of G protein deletion (rVSVΔG) [209]. The studies showed that rVSVΔG backbone is more attenuated and less immunogenic than rVSV and that the combination rVSVΔG with RSV-G antigen was not efficient for immunization. A non-propagating recombinant VSV bearing a deletion in the membrane-proximal domain of its G protein (VSV-G^STEM^) was used to vector RSV-F and G proteins and tested in vivo as an intranasal or intramuscular vaccine [210]. 

VSV has also been used to express the HMPV-F protein in a study aimed to design an enzyme-linked immunosorbent assay (ELISA) specific for HMPV [21]. The study showed that it is possible to efficiently express HMPV-F protein from the 2^nd^ genomic position of the VSV vector. The examples of VSV-based chimeric vaccines are described in Table 5. 

### 4.6. Recombinant Chimeric HMPV

The only chimeric vaccine against HMPV advanced to clinical trials so far is a recombinant HMPV virus rendered less-infectious as a result of replacing its P ORF with the counterpart of a closely-related AMPV_C [116]. This strategy of attenuation is based on the restriction of replication in vivo based on the host incompatibility of some of the viral components. In the first study, an HMPV chimera with either N (rHMPV-N_A_) or P (rHMPV-P_A_) gene exchanged for its AMPV_C counterpart was tested as a vaccine in hamsters and AGMs (Table 6). The N and P genes of AMPV_C share 75 and 68% nucleotide sequence identity with their HMPV counterparts, which suggested good integration of heterologous proteins into the HMPV particle [4,211]. rHMPV-N_A_ and rHMPV-P_A_ viruses replicated more efficiently in vitro, compared to their parental HMPV strain. When tested in vivo in hamsters, the replication of both chimeric viruses was decreased 100-fold, and rHMPV-N_A_ was slightly more attenuated than rHMPV-P_A_. Both chimeras were equally immunogenic and protected the vaccinated animals from wt HMPV infection. When tested in AGMs, rHMPV-P_A_ was more attenuated than rHMPV-N_A_ and was almost equally immunogenic, and both chimeras conferred protection against wt HMPV [116]. This study identified the more attenuated HMPV-P_A_ chimera as a more promising vaccine candidate for further development. The rHMPV-P_A_ virus has been recently tested as a vaccine in phase 1 clinical trial sequentially in adults, HMPV-seropositive and HMPV-seronegative children (NCT01255410) [25]. The trial found rHMPV-P_A_ virus being appropriately restricted in adults and seropositive children, yet insufficiently infectious and immunogenic in HMPV-seronegative children, thus leaving no other candidates for an anti-HMPV vaccine in clinical trials. 

## 5. Potential Development of Chimeric Vaccines against HMPV

As described, many studies have been performed with viral vectors (mainly paramyxoviruses) expressing pneumoviral proteins. Although the majority of those studies have focused on the development of anti-RSV vaccines, their results are an indicator of a potential for a successful vaccine candidate against HMPV. The efficacy of chimeric vaccines against HMPV can be potentially improved in many ways. 

The immunogenicity of HMPV-F protein could be increased by codon optimization for human expression. This modification is based on the degeneracy of the genetic code and makes it possible to optimize the sequence in order to improve its expression in a specific expression system, for example, in human cells. Codon optimization along with amino acid substitutions that rendered the RSV-F amino acid sequence identical to the early passage of the original A2 isolate (HEK-substitutions) [111] improved the immunogenicity of RSV-F protein expressed by rB/HPIV3 by 5-fold, but not the protection against wt RSV infection [66]. Codon optimization alone conferred a 2.1-fold increase in RSV-F expression, and HEK-substitutions alone conferred a 2.4-fold increase. 

Although exogenous proteins are usually well-expressed on the surface of the cells infected by chimeric viruses, efficient incorporation of foreign proteins into the viral particles remains challenging. This can be achieved by TMCT domain swapping between the insert and the vector, as it has been demonstrated for the rB/HPIV3//RSV-F^2nd^ virus [67]. Swapping of either the CT domain or TMCT domains increased the packaging efficiency 19–20-fold, reaching the level of RSV-F packaging into wt RSV virions. RSV-F^(TMCT)^ insert did not influence the incorporation of vector’s HN protein but it decreased the packaging of its F protein by 50–60%. TMCT modification did not influence viral replication in vitro, but it resulted in 10^1^–10^2^- and 10^2^–10^3^-fold increase in attenuation in hamsters and rhesus macaques, respectively. Similar packaging improvement and restriction in hamsters were observed for rHPIV1-C^Δ170^ bearing RSV-F^(TMCT)^ at the 1^st^ or the 2^nd^ gene position [155]. Interestingly, neither CT [208] nor TMCT domain exchange [207] improved packaging efficiency of RSV-F into VSV-GP particles, yet TMCT modification was necessary to efficiently express surface proteins of other viruses, for example, HIV-1 [212,213]. This proves that exchanging TMCT domains could be an attractive strategy to increase the incorporation of HMPV antigens into vector’s particles, yet this modification can cause an additional attenuating effect on the chimeric virus and each combination of a vector with antigen requires individual design. 

It has been demonstrated that the immunization with rB/HPIV3 expressing both RSV-F and G was more efficient than simultaneous immunization with two single-insert chimeras bearing either RSV-F or G [61]. It is supposed that this effect is due to interactions between these two surface glycoproteins that are closely associated one to another when expressed simultaneously within the same infected cell. Considering the structural similarities between RSV and HMPV, it might be presumed that the simultaneous expression of both HMPV-F and G proteins could have a beneficial effect on the efficacy of the vaccine, as it was also demonstrated for AMPV_C [189]. However, the study performed on virus-like particles (VLPs) expressing HMPV-F alone or along with the G protein showed that co-incorporation of the HMPV-G protein into VLP does not improve the immunogenicity conferred by the F protein [84]. Therefore, it is not likely that coexpression of HMPV-F and G could improve the vaccine’s immunogenicity and other improvement strategies should be considered. 

Another possible strategy for improving the design of anti-HMPV vaccines could be the expression of more than one copy of HMPV antigen. The HMPV virus expressing two additional copies of its F gene and one additional copy of its G gene from the 1^st^ genomic position has been described [130]. The expression of F and G protein mRNA by this recombinant HMPV was increased more than 6-fold and 14-fold, respectively, compared to wt HMPV. The in vitro replication of this recombinant virus was approximately 7-times reduced comparing to wt HMPV, which is a relatively small reduction, considering the fact that the genome length of the virus was increased by 30%. This study showed that adding additional copies of the ORF of HMPV-F or/and G could potentially increase the expression of its antigens also in the backbone of other vectors. 

## 6. Conclusions

HMPV and RSV are important pediatric pathogens. Despite many years of research, no vaccine against either HMPV or RSV is currently available, underlining the need for novel solutions for vaccine design and/ or optimization of existing strategies. Generation of recombinant chimeric vaccines that protect against more than one pathogen is an advantageous and practical solution for elaborating a promising vaccine candidate. Many studies were performed on vaccines containing an antigen of RSV expressed by a heterologous virus, mainly the F protein, and some of these bivalent vaccines were advanced to clinical trials. Although the possibilities of generating chimeric vaccines against HMPV were less-explored than for RSV, numerous similarities between the two viruses can serve as a guide for the design of anti-HMPV vaccines. The antigens of HMPV and RSV were successfully expressed by various viral vectors, most of which belong to the Paramyxoviridae family. Notwithstanding that paramyxoviruses can readily express exogenous proteins from an added gene in their genome, many different factors condition the viability of a chimeric virus, the efficacy of exogene expression and the immunogenicity of a vaccine candidate. Not only does the choice of vector virus need be taken into consideration, various other aspects, namely, the positioning of an exogene in the vector’s genome, the size and the type of an insert, and the possibility of integration of a foreign protein into the vector’s particle, should be thoroughly considered. Numerous studies on the engineering of chimeric viruses indicate that each combination exogene–vector must be carefully designed and individually verified. Recombinant vaccine candidates against either HMPV or RSV that have been designed up to date indicate that using another virus to express HMPV antigens, or vice versa, can make it possible to overcome some major hurdles in HMPV vaccine development and obtain a good balance between immunogenicity and attenuation of a live recombinant vaccine. 

## Figures and Tables

**Table 1:** Examples of BPIV3-based vaccines against either RSV or HMPV with their immunogenicity and protection in animal models.

Chimeric Vaccine		Immunization		Increase in Serum Antibody Titers Post Immunization		Challenge	Challenge Virus Titers in RT [log_10_ PFU/g ± SE]		Reference
Vector	Insert	Animal Model	Dose	Virus ^A^-Neutralizing ^B^	IgG ELISA Titers ^C^		URT	LRT	
rB/HPIV3	RSV-F^1st^	hamsters	1 × 10^6^ TCID_50_	9.3 ± 0.5, 26 dpi	9.4, 26 dpi	1 × 10^6^ PFU of RSV, 28 dpi	2.9 ± 0.4, 5 dpc	2.1 ± 0.2, 5 dpc	[62]
	RSV-G^1st^			10.0 ± 0.3, 26 dpi	6.5, 26 dpi		1.9 ± 0.2, 5 dpc	≤1.7, 5 dpc	
	RSV_A_-F^1st^	Rhesus macaques	2 ^D^ × 10^5^ TCID_50_	7.3 ± 0.0, 27 dpi	8.0, 27 dpi	No challenge was performed			[63]
	RSV_A_-G^1st^			7.3 ± 1.4, 27 dpi	5.5, 27 dpi				
	RSV_A_-F^1st^ G^1st^			8.8 ± 1.0, 27 dpi	8.0 (anti-F), 5.5 (anti-G), 27 dpi				
	RSV_A_-F^1st^ + ^H^ RSV_A_-G^1st^			7.3 ± 0.8, 27 dpi	5.5 (anti-F), 5.5 (anti-G), 27 dpi				
	RSV_B_-F^1st^			6.8 ± 0.5, 27 dpi	4.0, 27 dpi				
	RSV_B_-G^1st^			7.8 ± 1.0, 27 dpi	5.5, 27 dpi				
	RSV_B_-F^1st^ G^1st^			7.3 ± 0.8, 27 dpi	1.5 (anti-F), 5.5 (anti-G), 27 dpi				
	RSV-F^1st^	hamsters	1 × 10^6^ TCID_50_	10.1 ± 0.2, 28 dpi	nd	1 × 10^6^ PFU of RSV, 31 dpi	3.4 ± 0.2, 3 dpc	2.9 ± 0.2, 3 dpc	[148]
	RSV-F^2nd^			10.7 ± 0.3, 28 dpi			3.1 ± 0.1, 3 dpc	3.0 ± 0.2, 3 dpc	
	RSV-F^3rd^			10.4 ± 0.2, 28 dpi			3.6 ± 0.2, 3 dpc	≤2.7, 3 dpc	
	RSV-F^6th^			11.0 ± 0.4, 28 dpi			4.0 ± 0.2, 3 dpc	3.1 ± 0.3, 3 dpc	
BPIV3	RSV_A_-F^6th^ G^6th^		1 × 10^6^ PFU	5.4 ± 0.7, 21 dpi		1 × 10^6^ PFU of RSV, 21 dpi	1.7 ± 0.5, 4 dpc	1.4 ± 0.5, 4 dpc	[163]
rB/HPIV3	RSV-F^1st^		1 × 10^6^ PFU	5.5 ± 0.5, 28 dpi		1 × 10^6^ PFU of RSV, 28 dpi	<0.8 ± 0.1, 4 dpc	<0.5 ± 0.0, 4 dpc	[149]
	RSV-F^2nd^			6.9 ± 0.7, 28 dpi			<1.3 ± 0.6, 4 dpc	<1.6 ± 1.0, 4 dpc	
	RSV-G^1st^			3.4 ± 0.5, 28 dpi			<1.0 ± 0.3, 4 dpc	<0.7 ± 0.1, 4 dpc	
	RSV-G^2nd^			3.4 ± 0.5, 28 dpi			<0.8 ± 0.1, 4 dpc	<0.8 ± 0.3, 4 dpc	
	HMPV-F^1st^			7.8 ± 1.0, 28 dpi		1 × 10^6^ PFU of HMPV, 28 dpi	3.5 ± 0.8, 4 dpc	<0.5 ± 0.2, 4 dpc	
	HMPV-F^2nd^			7.4 ± 1.0, 28 dpi			<0.9 ± 0.4, 4 dpc	<0.5 ± 0.1, 4 dpc	
	HMPV-F^2nd^	AGMs	6.4 × 10^5^ PFU	7.1 ± 1.2 (HMPV_A), 2.7 ± 1.1 (HMPV_B), 28 dpi		5 × 10^5^ PFU of HMPV, 28 dpi	2.3 ± 1.1 ^E^	<1.3 ± 0.0 ^F^	[122]
	RSV-F^2nd^		2^D^ × 2–3 × 10^5^ PFU	4.0 ± 1.0 (RSV_A), 3.4 ± 1.8 (RSV_B), 28 dpi	8.2, 28 dpi	7 × 10^5^ PFU of RSV, 28 dpi	<1.2 ± 0.4 ^E^	<1.2 ± 0.3 ^G^	[121]
	RSV-F^2nd(SOL)^			4.1 ± 1.5 (RSV_A), 4.6 ± 1.4 (RSV_B), 28 dpi	8.0, 28 dpi		<1.1 ± 0.2 ^E^	<1.1 ± 0.0 ^G^	

^A^ HMPV or RSV, depending on the antigen inserted; ^B^ mean reciprocal log_2_-fold increase in 60% plaque reduction neutralization assay (PRNT60) ± standard error of the difference (SE); ^C^ log_2_-fold increase in serum immunoglobulin G (IgG) titers determined by ELISA; ^D^ simultaneous immunization intranasally and intratracheally; ^E^ daily mean peak titers from 0–11 dpc; ^F^ daily mean peak titers collected on 0, 2, 4, 6, and 8 dpc; ^G^ daily mean peak titers collected on 1, 3, 5, 7, and 9 dpc; ^H^ animals were immunized with a 10^5^ TCID_50_ mixture of the two viruses; SOL: soluble protein lacking TMCT domains; RT: respiratory tract; URT and LRT: upper and lower RT, respectively; AGMs: African green monkeys; nd: not determined; dpi: days post the last immunization; dpc: days post challenge.

**Table 2:** Examples of HPIV1-based vaccines against either RSV or HMPV with their immunogenicity and protection in animal models.

Chimeric Vaccine		Immunization		Increase in Serum Virus ^A^-Neutralizing Antibody Titers Post Immunization ^B^	Challenge	Challenge Virus Titers in RT [log_10_ PFU/g ± SE]		Reference
Vector	Insert	Animal Model	Dose			URT	LRT	
HPIV1	HMPV-F^1st^	hamsters	1 × 10^6^/10^6.4^ TCID_50_^D^	8.3 ± 0.4, 26 dpi	1 × 10^5.7^ TCID_50_ of RSV, 28 dpi	3.3 ± 0.2 ^C^, 4 dpc	≤1.5 ± 0.0 ^C^, 4 dpc	[54]
	HMPV-SH^3rd^			≤2.9 ± 0.0, 26 dpi		5.3 ± 0.2 ^C^, 4 dpc	2.7 ± 0.1 ^C^, 4 dpc	
	HMPV-G^3rd^		1 × 10^6^/10^7.4^ TCID_50_^D^	≤2.9 ± 0.0, 26 dpi		4.6 ± 0.5 ^C^, 4 dpc	2.4 ± 0.4 ^C^, 4 dpc	
	HMPV_A_-F^1st^		1 × 10^5^ TCID_50_	6.0 ± 0.8 (HMPV_B), 8.6 ± 0.2 (HMPV_A), 33 dpi	1 × 10^5.5^ TCID_50_ of HMPV_A or B, 50 dpi	2.9 ± 0.3 (HMPV_B), 3.9 ± 0.1 (HMPV_A), 4 dpc	nd	[6]
rHPIV1- LY942A	RSV-F^1st^			<3.3, 28 dpi	1 × 10^6^ PFU of RSV, 30 dpi	7.0, 3 dpc	6.1, 3 dpc	[145]
	RSV-F^2nd^			<3.3, 28 dpi		6.9, 3 dpc	4.9, 3 dpc	
	RSV-F^3rd^			<3.3, 28 dpi		6.7, 3 dpc	6.0, 3 dpc	
rHPIV1- CΔ170	RSV-F^1st^			7.3 ± 0.3, 28 dpi		4.8, 3 dpc	3.7, 3 dpc	
	RSV-F^2nd^			4.7 ± 0.7, 28 dpi		6.2, 3 dpc	4.3, 3 dpc	
	RSV-F^3rd^			6.7 ± 0.8, 28 dpi		5.5, 3 dpc	4.3, 3 dpc	
	RSV-F^1st(PF)^		1 × 10^6^ TCID_50_	9.58/ 4.87 ^E^, 28 dpi		4.47, 3 dpc	3.04, 3 dpc	[155]
	RSV-F^2nd(PF)^			6.90/ 2.58 ^E^, 28 dpi		4.81, 3 dpc	4.16, 3 dpc	
	RSV-F^1st(PF,TMCT)^			6.08/ 3.33 ^E^, 28 dpi		4.76, 3 dpc	4.04, 3 dpc	
	RSV-F^2nd(PF,TMCT)^			4.38/ 2.53 ^E^, 28 dpi		5.38, 3 dpc	4.65, 3 dpc	

^A^ HMPV or RSV, depending on the antigen inserted; ^B^ mean reciprocal log_2_-fold increase in PRNT60 ± SE; ^C^ log_10_TCID_50_/g ± SE; ^D^ the dose of the prime/boost immunization; ^E^ values measured with/without added guinea pig complement; RT: Respiratory tract; URT and LRT: upper and lower RT, respectively; PF: pre-fusion form of RSV-F; TMCT: chimeric form of RSV-F where transmembrane (TM) and cytoplasmic (CT) domains were exchanged for their counterparts from the vector; nd: not determined; dpi: days post the last immunization; dpc: days post challenge.

**Table 3:** Examples of SeV-based vaccines against either RSV or HMPV with their immunogenicity and protection in animal models.

Chimeric Vaccine		Immunization		Virus ^A^-Neutralization by Diluted Sera from Vaccinated Animals	Challenge	Challenge Virus Titers in LRT	Reference
Vector	Insert	Animal Model	Dose				
SeV	RSV-G^5th^	hamsters	2 × 10^8^ PFU	50–60% ^B^, 14 dpi	10^6^ PFU of RSV, 28 dpi	<dl ^E^	[173]
	RSV-G^5th^		2 × 10^6^ PFU	58 ± 12% (RSV_A) ^B^, 35 ± 19% (RSV_B) ^B^, 28 dpi	7.5 × 10^6^ PFU of RSV_A, 35 dpi	10^3^–10^4^ PFU/rat, 3 dpc	[152]
	RSV-F^5th^			82 ± 7% (RSV_A) ^B^, 84 ± 12% (RSV_B) ^B^, 28 dpi		10^3^–10^4^ PFU/rat, 3 dpc	
					1.5 × 10^6^ PFU of RSV_A or B, 35 dpi	<dl, 3 dpc	
	RSV-F^5th(SOL)^			>80% (RSV_A) ^B^, >80% (RSV_B) ^B^, 28 dpi	1.5 × 10^6^ PFU of RSV, 35 dpi	<dl, 3 dpc	[175]
	RSV-F^5th^	AGMs	1 × 10^6^ EID_50_	9.7 ^C^, 25 dpi	1.4 × 10^6^ PFU of RSV, 28 dpi	<dl, 3 dpc	[151]
	HMPV-F^5th(TR)^	cotton rats	2 × 10^6^ TCID_50_	~200 IC_50_^D^, 28–42 dpi (HMPV_A)	2 × 10^5^– 3 × 10^6^ TCID_50_ of HMPV, 28–42 dpi	10^2^–10^3^ TCID_50_/lung, 4 dpc	[153]

^A^ HMPV or RSV, depending on the antigen inserted; ^B^ mean % plaque reduction by sera diluted 1:64; ^C^ mean reciprocal log_2_-fold increase in PRNT60; ^D^ serum dilution at which 50% of virus infection was inhibited (IC_50_); ^E^ mean virus titers from 3, 5, 7, and 10 dpc; LRT: lower respiratory tract; SOL: soluble version of the antigen; TR: truncated protein; AGMs: African green monkeys; EID_50_: 50% effective infectious dose in eggs; dpi: days post the last immunization; dpc: days post challenge; <dL: below the detection limit, not detected.

**Table 4:** Examples of NDV-based vaccines against either RSV or AMPV with their immunogenicity and protection in animal models.

Chimeric Vaccine		Immunization		Sera positive for virus ^A^-Neutralizing antibodies ^B^	Challenge	Challenge Virus in LRT	Reference
Vector	Insert	Animal Model	Dose				
NDV	RSV-F^3rd^	BALB/c mice	5 × 10^5^ PFU	nd	1 × 10^7^ PFU of RSV, 28 dpi	1 × 10^4^ PFU/g, 5 dpc	[185]
	AMPV_C_-G^5th^	turkeys	1 × 10^6^ TCID_50_	40% ^C^	4.2 × 10^3^ ID_50_ of AMPV_C, 14 dpi	100%/90%/70% ^E^	[187]
			2^D^ × 10^6^ TCID_50_	40%/50%^C^		100%/100%/80% ^E^	
	AMPV_A_-G^5th^		1 × 10^6^ TCID_50_	nd	1 × 10^2^ ID_50_ of AMPV_A or B, 14 dpi	100%/100%/30% ^F^	[188]
	AMPV_B_-G^5th^					100%/100%/50% ^F^	
	AMPV_C_-G^5th^		1 × 10^6^ TCID_50_	40% ^C^		100%/100%/80% ^E^	[189]
	AMPV_C_-G^5th^ F^5th^			70% ^C^		100%/100%/60% ^E^	
	AMPV_C_-G^5th^		2 ^D^ × 10^6^ TCID_50_	40%/50% ^C^		100%/90%/70% ^E^	
	AMPV_C_-G^5th^ F^5th^			60%/100% ^C^		100%/80%/20% ^E^	

^A^ AMPV_C or RSV, depending on the antigen inserted; ^B^ determined by ELISA; ^C^ % of immunized birds that seroconverted at 14 dpi (after single immunization) or at 14/28 dpi (after prime/boost immunization); ^D^ prime and boost immunizations in 2-weeks interval; ^E^ % of birds with detected challenge virus RNA shedding, results for 3/5/7 dpi; ^F^ % of birds with detected challenge virus RNA shedding, results for 5/7/9 dpc; ID_50_: 50% infective dose; LRT: lower respiratory tract; dpi: days post immunization; dpc: days post challenge.

**Table 5:** Examples of VSV-based vaccines against either RSV or AMPV with their immunogenicity and protection in animal models.

Chimeric Vaccine		Immunization		Serum Neutralizing Antibody Titers Post Immunization	RSV ELISA Titer ^B^	Challenge	Challenge Virus Titers in RT		Reference
Vector	Insert	Animal Model	Dose				URT	LRT	
VSV	RSV-F^4th^	BALB/c mice	2 ^D^ × 10^4^ PFU	1:32 ^A^	4.096	1.2 × 10^5^ PFU of RSV, 28 dpi	<50 PFU/ mL, 4 dpc	<50 PFU/mL, 4 dpc	[209]
	RSV-G^4th^			1:16 ^A^	128		<50 PFU/ mL, 4 dpc	<50 PFU/mL, 4 dpc	
rVSVΔG	RSV-F^4th^		3 ^E^ × 1.25 × 10^3^ PFU	<1:8 ^A^	1.024		<50 PFU/ mL, 4 dpc	<50 PFU/mL, 4 dpc	
	RSV-G^4th^		3 ^E^ × 10^4^ PFU	<1:8 ^A^	<64		1 × 10^4.2^ PFU/ mL, 4 dpc	1 × 10^5.4^ PFU/mL, 4 dpc	
rVSV-GP	RSV-F^5th CO^		1 × 10^7^ TCID_50_	~6.9 log_2_ IC_50_^G^	nd	1 × 10^6^ PFU of RSV, 28 dpi	nd	~4.45 log_10_ RSV copies/µg RNA, 5 dpc	[207]
			3 ^F^ × 10^7^ TCID_50_	~6.53/~9.5/~8.68 ^H^ log_2_ IC_50_^G^				~5.4 log_10_ RSV copies/µg RNA, 5 dpc	
rVSV-G^STEM^	RSV-F^1st^		2 × 10^7^ PFU ^I^	prime: 3.2/1.8; boost: 3.8/3.0 (RSV_A); 2.4 (RSV_B) ^J^		1 × 10^6^ PFU of RSV_A or 2 × 10^5^ PFU of RSV_B, 28 dpi	~1 × 10^1^ PFU/g, 100%^M^	1 × 10^0^–10^1^ PFU/g, 100% ^M^	[210]
	RSV-F^3rd^			prime: 3.3/1.9; boost: 3.4/3.0 (RSV_A); 2.7 (RSV_B) ^J^			~1 × 10^1^ PFU/g, 100%^M^	1 × 10^0^–10^1^ PFU/g, 100% ^M^	
	RSV-F^1st^		1 × 10^6^ PFU ^K^	2.19/1.34 ^L^		1 × 10^6^ PFU of RSV_A, 28 dpi	1 × 10^2^–10^3^ PFU/g, 0% ^M^	1 × 10^3^ PFU/g, 70% ^M^	
	RSV-F^3rd^			2.65/1.46 ^L^			1 × 10^1^–10^2^ PFU/g, 10% ^M^	1 × 10^2^–10^3^ PFU/g, 90% ^M^	
	RSV-F^5th^			2.63/1.62 ^L^			1 × 10^1^–10^2^ PFU/g, 0% ^M^	1 × 10^2^–10^3^ PFU/g, 80% ^M^	
	RSV-F^3rd^		1 × 10^5^ PFU	4.68 (anti-F), <2.0 (anti-G)			1 × 10^2^–10^3^ PFU/g, 0% ^M^	1 × 10^1^–10^2^ PFU/g, 40% ^M^	
	RSV-G^3rd^			<2.0 (anti-F), 4.42 (anti-G)			~1 × 10^3^ PFU/g, 0% ^M^	1 × 10^1^–10^2^ PFU/g, 50% ^M^	
	RSV-F^3rd^ and G^3rd^		1 × 10^5^ PFU of the two viruses	4.55 (anti-F), 4.58 (anti-G)			1 × 10^2^–10^3^ PFU/g, 0% ^M^	1 × 10^0^–10^1^ 1 PFU/g, 100% ^M^	

^A^ the last serum dilution in which RSV CPE was not detected, measured at 14 dpi; ^B^ dilution of pooled mouse serum which corresponded to an OD_450_ of 0.5; ^C^ viral RNA shedding in LRT of the birds; results for 3/5/7 dpi; ^D^ prime and boost immunizations in 2-week intervals; ^E^ prime and two boost immunizations in 2-week intervals; ^F^ mice were immunized 3 times by intramuscular injection in 4-week intervals; ^G^ serum dilution, at which 50% of virus infection was inhibited (IC50); ^H^ values determined after the prime immunization/1st boost/2nd boost, i.e., 28/56 dpi; ^I^ prime and boost immunizations by intramuscular injection in 4-week intervals; ^J^ values log_10_PRNT_60_ measured with/ without added guinea pig complement 28 dpi and 28 dp boost immunization. Values for anti RSV_B antibodies were determined only without the complement 28 dp boost immunization; ^K^ immunizations by intramuscular injection with no boost; ^L^ values log_10_PRNT_60_ measured with/ without added guinea pig complement 28 dpi; ^M^ % of vaccinated mice that were protected against RSV 4 days post challenge; RT: Respiratory tract; URT and LRT: upper and lower RT; CO: codon-optimized; dpi: days post the last immunization; dpc: days post challenge.

**Table 6:** The results of preclinical trials of rHMPV-N_A_ and rHMPV-P_A_ in hamsters and AGMs.

Chimeric Vaccine	Immunization		HMPV-Neutralizing Antibody Titers Prior to Challenge [log_2_± SE]	Challenge	Mean Peak HMPV Titer in RT		Reference
	Animal Model	Dose			URT	LRT	
rHMPV-N_A_	hamsters	1 × 10^5.7^ PFU	5.6 ± 0.6, 27 dpi	1 × 10^5.7^ PFU of HMPV, 28 dpi	≤1.5, 0% ^A^, 3 dpc	≤1.5, 0% ^A^, 3 dpc	[116]
rHMPV-P_A_			4.9 ± 0.6, 27 dpi		≤1.5, 0% ^A^, 3 dpc	≤1.5, 0% ^A^, 3 dpc	
rHMPV-N_A_	AGMs	2 ^B^ × 10^6^ PFU	5.4 ± 0.4, 28 dpi	2 ^B^ × 10^6^ PFU of HMPV, 28 dpi	<0.7, 0% ^C^	<0.7, 0% ^C^	
rHMPV-P_A_			5.0 ± 0.5, 28 dpi		<0.7, 0% ^C^	<0.7, 0% ^C^	

^A^ mean HMPV titer log_10_ PFU/g of tissue, % of animals with detectable challenge virus; ^B^ simultaneous inoculation intranasally and intratracheally; ^C^ samples collected on 2, 4, 6, 8 dpc, virus titers determined by plaque assay with detection limit 0.7 log_10_ PFU/ ml, % of animals with detectable challenge virus; RT: Respiratory tract; URT and LRT: upper and lower RT, respectively; AGMs: African green monkeys; dpi: days post the last immunization; dpc: days post challenge.
